# Connecticut

**Published:** 1897-08

**Authors:** 


*Connecticut.*—The Legislature of this State has wiped out the commission of three to enforce the law relative to bovine tuberculosis, and Governor Crook has appointed ex-State Senator William B. Sprague as Commissioner on Diseases of Domestic Animals. Mr. Sprague is a member of the Board of Agriculture and Vice-President of the Connecticut Dairymen's Association.

Section 211, amending Section 3671, relating to glanders, so that when an agent of the Humane Society finds a case of glanders or farcy he may call in a veterinary surgeon at the society's expense. If they agree that the animal is infected, the agent shall notify the owner, and unless the owner calls in a veterinarian within forty-eight hours, he must separate the animal from all others, except cattle, for ten days. If he still refuses to call a veterinarian, the agent and veterinarian of the Humane Society shall make a second examination, and if they agree that the animal is infected, the Humane Society may order its destruction. But if the owner calls a veterinarian and he disagrees with the Humane Society's veterinarian, a majority shall decide, and if infected the animal shall be destroyed at the owner's expense. The expense of the veterinarian called by the owner shall be paid for by him, and the expense of the third veterinarian shall be paid by the State.

**Those who failed to view the World's Fair should not miss the Nashville exposition.**

The glanders law further provides that no veterinarian shall be employed unless a graduate of a regular school and of two years' practice in the State.

Section 199 repeals the tuberculosis law of 1895, and provides that any person bringing cattle into the State shall within six days notify the Commissioner on Domestic Animals. When any disease exists among domestic animals in this State, the commissioner may quarantine all affected animals and prohibit the sale of their products, "but no animal shall be quarantined that does not give evidence of disease by competent physical examination, and no animal shall be quarantined more than thirty days." If the commissioner decides that quarantined animals should be killed, he may cause them to be killed after their value has been adjusted by the commissioner and their owner, and if they cannot agree, a third person may be called in, and the value determined upon shall be paid by the State. "But no animal whose physical condition indicates that it is of no real value, and no animal that has not been in this State for six months prior to its quarantine shall be paid for by the State." The commissioner shall inspect animals at the request of the owner by physical examination; if free from disease "dangerous to the public health" he shall so certify to the owner. It is made the duty of selectmen to report to the commissioner animals "infected with contagious disease." The Governor shall biennally appoint a commissioner who shall be "a practical farmer and stock-breeder of at least ten years' experience." His salary is fixed at $1500 and expenses, and he may appoint assistants with the approval of the Governor.

**Those who viewed the splendor of the famous "White City" will want to see Nashville.**

The bill destined to require registration of all practitioners in the State and the establishment of a State Board of Examiners, was defeated in the lower branch of the Legislature.

This State has passed a law intended to prevent the spreading of contagious diseases, by requiring those who bring cattle from any other State to notify the Commissioner on Domestic Animals thereof, and to designate the true physical condition of such animals. A violation is punishable by a fine of not more than fifty dollars. The commissioner has the right to quarantine any animals which he considers are affected with any contagious disease. A competent physical examination is required before the quarantine may be enforced, which may not last longer than thirty days. Should the commissioner consider any of the animals endangering public health, he may order them killed, but not before the price of said animal has been fixed satisfactorily to the owner and commissioner, when said price shall be paid to the owner by the State. An animal whose physical condition shows it to be of no value, or one which has not been in the State six months prior to its condemnation, shall not be paid for by the State. It is the duty of the selectman of each town to report to the commissioner all cases of contagious diseases. The commissioner is appointed by the Governor for the term of two years, and the salary is fifteen hundred dollars a year. All acts inconsistent with this one are now repealed.

